# Inhibition of EZH2 by chidamide exerts antileukemia activity and increases chemosensitivity through Smo/Gli-1 pathway in acute myeloid leukemia

**DOI:** 10.1186/s12967-021-02789-3

**Published:** 2021-03-21

**Authors:** Xuejie Jiang, Ling Jiang, Jiaying Cheng, Fang Chen, Jinle Ni, Changxin Yin, Qiang Wang, Zhixiang Wang, Dan Fang, Zhengshan Yi, Guopan Yu, Qingxiu Zhong, Bing Z. Carter, Fanyi Meng

**Affiliations:** 1 Department of Hematology, Nanfang Hospital, Southern Medical University, Guangzhou, 510515 Guangdong China; 2 Department of Hematology, Kanghua Hospital, Dongguan, 523080 Guangdong China; 3 Section of Molecular Hematology and Therapy, Department of Leukemia, The University of Texas MD Anderson Cancer Center, Houston, TX USA

**Keywords:** EZH2, Chidamide, Leukemia, Chemosensitivity, Smo/Gli-1

## Abstract

**Background:**

Epigenetic dysregulation plays important roles in leukemogenesis and the progression of acute myeloid leukemia (AML). Histone acetyltransferases (HATs) and histone deacetylases (HDACs) reciprocally regulate the acetylation and deacetylation of nuclear histones. Aberrant activation of HDACs results in uncontrolled proliferation and blockade of differentiation, and HDAC inhibition has been investigated as epigenetic therapeutic strategy against AML.

**Methods:**

Cell growth was assessed with CCK-8 assay, and apoptosis was evaluated by flow cytometry in AML cell lines and CD45 + and CD34 + CD38- cells from patient samples after staining with Annexin V-fluorescein isothiocyanate (FITC)/propidium iodide (PI). EZH2 was silenced with short hairpin RNA (shRNA) or overexpressed by lentiviral transfection. Changes in signaling pathways were detected by western blotting. The effect of chidamide or EZH2-specific shRNA (shEZH2) in combination with adriamycin was studied in vivo in leukemia-bearing nude mouse models.

**Results:**

In this study, we investigated the antileukemia effects of HDAC inhibitor chidamide and its combinatorial activity with cytotoxic agent adriamycin in AML cells. We demonstrated that chidamide suppressed the levels of EZH2, H3K27me3 and DNMT3A, exerted potential antileukemia activity and increased the sensitivity to adriamycin through disruption of Smo/Gli-1 pathway and downstream signaling target p-AKT in AML cells and stem/progenitor cells. In addition to decreasing the levels of H3K27me3 and DNMT3A, inhibition of EZH2 either pharmacologically by chidamide or genetically by shEZH2 suppressed the activity of Smo/Gli-1 pathway and increased the antileukemia activity of adriamycin against AML in vitro and in vivo.

**Conclusions:**

Inhibition of EZH2 by chidamide has antileukemia activity and increases the chemosensitivity to adriamycin through Smo/Gli-1 pathway in AML cells (Fig. 5). These findings support the rational combination of HDAC inhibitors and chemotherapy for the treatment of AML.

**Supplementary Information:**

The online version contains supplementary material available at 10.1186/s12967-021-02789-3.

## Background

Acute myeloid leukemia (AML) is the most common adult hematological malignancy with a poor prognosis. Nearly 80% of patients achieve initial remission after induction chemotherapy, but most relapse and then fail to reinduction chemotherapy^1^. Treatment failure is associated with simultaneous resistance to chemotherapeutic drugs and survival of leukemia stem cells (LSCs) [2]. Recent studies have shown that constitutive activation of multiple pathways contributes to chemoresistance in AML [3, 4]. Epigenetic modification regulates various biological functions, such as histone acetylation and deacetylation, DNA methylation and demethylation^5^, ^6^. Epigenetic dysregulation plays important roles in leukemogenesis and progression, and targeted epigenetic regulation is a promising therapeutic strategy against AML [7, 8].

Histone acetyltransferases (HATs) and histone deacetylases (HDACs) reciprocally regulate the acetylation and deacetylation of nuclear histones [9]. Histone modification directly affects the nucleosome structure and activities of oncogenes and transcription factors [10]. Aberrant activation of HDACs regulates the expression of multiple genes and activities of signaling pathways, resulting in uncontrolled proliferation and blockade of differentiation related to leukemogenesis in myeloid malignancies [12, 13]. HDAC inhibitors suppress proliferation and induce apoptosis and have synergistic antileukemia effects in combination with cytotoxic agents in AML cells [14, 15]. Chidamide, a novel benzamide-type HDAC inhibitor, has been demonstrated to induce cell differentiation and apoptosis by specifically inhibiting HDAC1, HDAC2, HDAC3 and HDAC10 [16]. It was initially developed to treat T/B-cell lymphoma/leukemia, breast cancer and lung cancer [17–20]. Recent studies have shown that chidamide significantly inhibits the viability of AML cells and LSCs and increases the sensitivity to cytotoxic agents by disrupting multiple pathways, activating reactive oxygen species (ROS) and accumulating DNA damage [21–24]. Thus, HDAC inhibition provides a potential strategy to improve chemotherapeutic effects in AML. However, the mechanisms of action of chidamide are not fully understood.

It has been reported that inhibition of enhancer of zeste homolog 2 (EZH2) contributes to the antitumor effect of HDAC inhibitors in neuroblastoma cells and lung cancer cells [25, 26]. EZH2 is the functional core subunit of polycomb repressive complex 2 (PRC2) and plays a pivotal role in catalyzing the methylation of lysine 27 of histone H3 (H3K27) [27, 28]. Overexpression of EZH2 indicates a poor prognosis in patients with lymphoma, melanoma, or breast cancer, and EZH2 is considered as a potential therapy target in malignant tumors [29, 30]. EZH2 supports leukemogenesis by blocking cell differentiation in AML, and inhibition of EZH2 is an effective strategy to eliminate LSCs through Hedgehog pathway [31–33]. Smo/Gli-1 is the key component of signal transduction in Hedgehog pathway, which is closely associated with the chemoresistance of AML cells and survival of LSCs [34–36]. Disruption of the Smo/Gli-1 pathway has been demonstrated to improve chemotherapeutic effects in AML, and Smo inhibitors have been approved by the Food and Drug Administration (FDA) to treat AML patients in combination with chemotherapy [37–39]. A recent study showed that an EZH2 inhibitor increased the sensitivity to cytotoxic agent 5-fluorouracil through Smo/Gli-1 pathway in colorectal cancer [40]. Strategies to improve chemotherapeutic effects are also needed for the treatment of AML.

Our previous studies showed that EZH2 overexpression and activation of Smo/Gli-1 pathway were related to the poor prognosis in AML patients, and Smo inhibitor effectively decreased leukemia growth and increased chemosensitivity [41–43]. Chidamide has a promising antileukemia effect without clear mechanism in AML. In this study, we demonstrated that chidamide exerted antileukemia activity in AML cells and stem/progenitor cells and increased sensitivity to adriamycin in vitro and in vivo by inhibiting EZH2 through Smo/Gli-1 pathway.

## Materials and methods

### Cells

Kasumi-1 and HL-60/ADM cells (Institutes for Biological Sciences Cell Resource Center, Chinese Academy of Sciences, Shanghai, China) were cultured in RPMI-1640 medium (HyClone, USA) supplemented with 10% heat-inactivated fetal bovine serum (Gibco, USA) in a humidified atmosphere of 5% CO2 at 37 °C. Bone marrow samples were obtained from AML patients, except those with the M3 subtype, and healthy donors for stem cell transplantation after informed consent was obtained following approval by the institutional ethics committee at Nanfang Hospital in accordance with the Declaration of Helsinki. Mononuclear cells were purified by Ficoll-Hypaque (Sigma-Aldrich, USA) density gradient centrifugation and cultured in α-MEM supplemented with 10% fetal bovine serum. Table 1 summarizes the clinical characteristics of the patients.

**Table 1:** Characteristics of AML patients and experiments

Pt No	Source	% Blasts	Disease status	Molecular mutation	Cytogenetic	Experiments
1	BM	86	New diagnosis	IDH1 + TET2	46,XX	Chi
2	BM	71	New diagnosis	Negative	46,XY	Chi
3	BM	92	Relapse/refractory	*FLT3*-ITD	Complex	Chi
4	BM	65	New diagnosis	Negative	t(8;21)	Chi
5	BM	46	New diagnosis	Kit D816	t(8;21)	Chi
6	BM	77	Relapse	Negative	Complex	Chi + ADM
7	BM	63	New diagnosis	NPM1 + DNMT3a	46,XY	Chi + ADM
8	BM	90	New diagnosis	*FLT3*-ITD + NPM1	Complex	Chi + ADM
9	BM	74	New diagnosis	Negative	Inv(16)	Chi + ADM
10	BM	59	Relapse/refractory	*FLT3*-ITD + DNMT3a	46,XY	Chi + ADM
11	BM	81	Relapse	Negative	47,XX, + 8	Chi + ADM

*Pt No* Patient number, *BM* Bone marrow, *Chi* Chidamide, *ADM* Adriamycin

### Cell growth assay

Kasumi-1 and HL-60/ADM cells (2 × 10^5^ cells/ml) were plated in 96-well plates and treated with chidamide (Chipscreen Biosciences, China), adriamycin (MedChem Express, USA), or their combination. Cell growth was assessed with CCK-8 assay kit (Dojindo, Japan). After cells were incubated with 10 µL of CCK-8 solution for 2 h at 37 °C, the absorbance of each well was measured at 450 nm using a spectrophotometer (Thermo Fisher Scientific, USA). Cell viability was determined for the cells in each treated group and compared with that of untreated cells. The drug concentration resulting in 50% inhibition of cell growth (IC50) was calculated to evaluate the sensitivity of Kasumi-1 and HL-60/ADM cells to adriamycin.

### Flow cytometry analysis

Kasumi-1, HL-60/ADM cells (2 × 10^5^ cells/ml) and patient samples (5 × 10^5^ cells/ml) were treated with chidamide, adriamycin, or their combination. Cell apoptosis was estimated by flow cytometry (BD Biosciences, USA) after cells were stained with Annexin V-fluorescein isothiocyanate (FITC)/propidium iodide (PI) (NanJing KeyGen Biotechnology, China). Apoptosis in CD45 + and CD34 + CD38- cells was evaluated by flow cytometry (BD Biosciences, USA) after patient samples were incubated with anti-CD45-APC, anti-CD34-PC5.5 and anti-CD38-PE Cy7 antibodies (BD Biosciences, USA) and stained with Annexin V-FITC (NanJing KeyGen Biotechnology, China).

### EZH2 silencing and overexpression

A lentivirus carrying EZH2-specific short hairpin RNA (shRNA) and an EZH2-overexpressing lentivirus (LV-EZH2) were constructed by GeneChem (Shanghai, China). The targeting sequences for EZH2-specific shRNA (shEZH2) were as follows: shRNA-1, 5′-AACAGCTGCCTTAGCTTCA-3′; shRNA-2, 5′-AACAGCTCTAGACAACAAA-3′; shRNA-3, 5′-GGATAGAGAATGTGGGTTT-3′. The negative control for shEZH2 was a nontarget scrambled sequence: 5′-TTCTCCGAACGTGTCACGT-3′. Kasumi-1 and HL-60/ADM cells were transfected with lentivirus carrying enhanced green fluorescent protein (eGFP) and sorted by flow cytometry as described in our previous study [49]. The effects of EZH2 silencing or overexpression were confirmed by real-time polymerase chain reaction (RT-PCR) and western blotting. The Kasumi-1 and HL-60/ADM cells with the best EZH2 silencing efficacy and stable EZH2 overexpression were used in subsequent experiments.

### Western blotting analysis

Kasumi-1 and HL-60/ADM cells were treated with chidamide, adriamycin, or their combination, and cells were lysed in RIPA buffer (Sigma-Aldrich, USA). Protein levels were determined by western blotting as previously described [1, 44]. Briefly, whole-cell lysates were separated by SDS-PAGE and transferred to polyvinylidene difluoride (PVDF) membranes (Millipore, USA). Membranes were probed with an appropriate primary antibody and then incubated with a secondary antibody. The immunoblots were visualized using chemiluminescence horseradish peroxidase substrate (Millipore, USA) and analyzed with the Odyssey Infrared Imaging System (LI-COR Biosciences, USA). Antibodies against acetyl-histone H3 (#8173), DNMT3A (#3598), H3K27me3 (#9733), EZH2 (#5246), Smo (#4940), Gli-1 (#2643), AKT (#4685), p-AKT (#9614) and GAPDH (#5174) were purchased from Cell Signaling Technology (Beverly, MA, USA). Horseradish peroxidase-conjugated goat anti-mouse IgG and goat anti-rabbit IgG were obtained from Santa Cruz Biotechnology (Santa Cruz, CA, USA). GAPDH was used as a loading control.

### In vivo studies

Animal experiments were performed in accordance with protocols approved by the Nanfang Hospital Animal Care and Use Committee. Kasumi-1 or shEZH2 Kasumi-1 cells (1 × 10^7^) were injected subcutaneously into the right posterior flank of BALB/c nude mice. When the tumor size reached 150–200 mm^3^, the Kasumi-1 tumor-bearing mice were randomized to the following treatment groups (n = 10/group): vehicle control, adriamycin (3 mg/kg/d) by intraperitoneal injection, chidamide (12.5 mg/kg/d) by oral gavage or adriamycin plus chidamide for 7 days. Mice with shEZH2 Kasumi-1 neoplasms were randomized to the following treatment groups (n = 10/group): vehicle control and adriamycin (3 mg/kg/d) by intraperitoneal injection for 7 days. Tumor volume was calculated as V = 0.5 × longest × shortest^2^. Three mice in each group were sacrificed after treatment, and the tumors were weighed and fixed in 10% neutral formalin overnight. Histopathological and immunohistochemical examinations were performed to determine the protein expression of EZH2, Smo, Gli-1, and p-AKT in the tumor tissues.

### Statistical analysis

Cell experiments were conducted in triplicate, and data are expressed as the mean ± SEM. Statistical analyses were performed using a two-tailed Student’s t-test or one-way analysis of variance (ANOVA) for comparisons of multiple groups. *P* < 0.05 was defined as statistically significant.

## Results

### Chidamide, a novel HDAC inhibitor, suppresses growth and induces apoptosis in AML cells and stem/progenitor cells

**Fig. 1:**
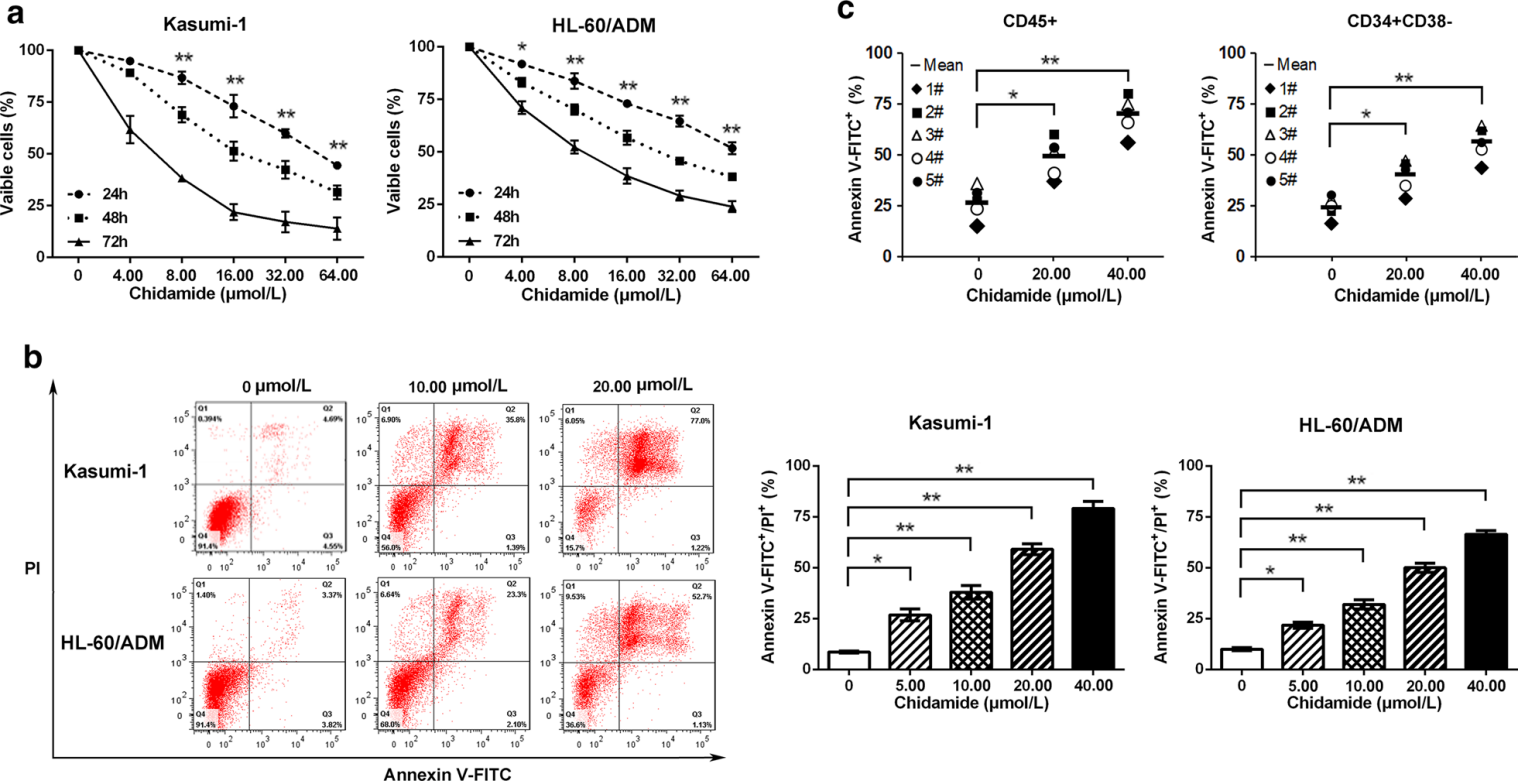
Chidamide suppresses growth and induces apoptosis in AML cells and stem/progenitor cells. **a** Kasumi-1 and HL-60/ADM cells were treated with chidamide for 24, 48 and 72 h. Cell viability was assessed with CCK-8. **b** Kasumi-1 and HL-60/ADM cells were treated with chidamide for 48 h. Apoptosis was determined by flow cytometry. **c** Patient samples were treated with chidamide for 48 h. Apoptosis in CD45 + and CD34 + CD38- cells was measured by flow cytometry. ^*^P < 0.05: ^**^P < 0.01

Kasumi-1, HL-60/ADM and primary AML cells were treated with HDAC inhibitor chidamide. Cell viability was determined with CCK-8 assay, and apoptosis was evaluated by flow cytometry after Annexin-FITC/PI staining. Chidamide markedly inhibited cell growth in Kasumi-1 and HL-60/ADM cells after treatment for 24, 48 or 72 h (Fig. 1a). Apoptosis was obviously induced in Kasumi-1 and HL-60/ADM cells after treatment with 5.00, 10.00, 20.00 or 40.00 μmol/L chidamide for 48 h (Fig. 1b). We also observed apoptosis in CD45 + and CD34 + CD38- stem/progenitor cells from AML patients after treatment with 20.00 or 40.00 μmol/L chidamide for 48 h (Fig. 1c), which had limited cytotoxicity in normal CD45 + and CD34 + cells from healthy donors (Additional file 1: Fig. S1).

### Chidamide sensitizes AML cells and stem/progenitor cells to adriamycin

**Fig. 2:**
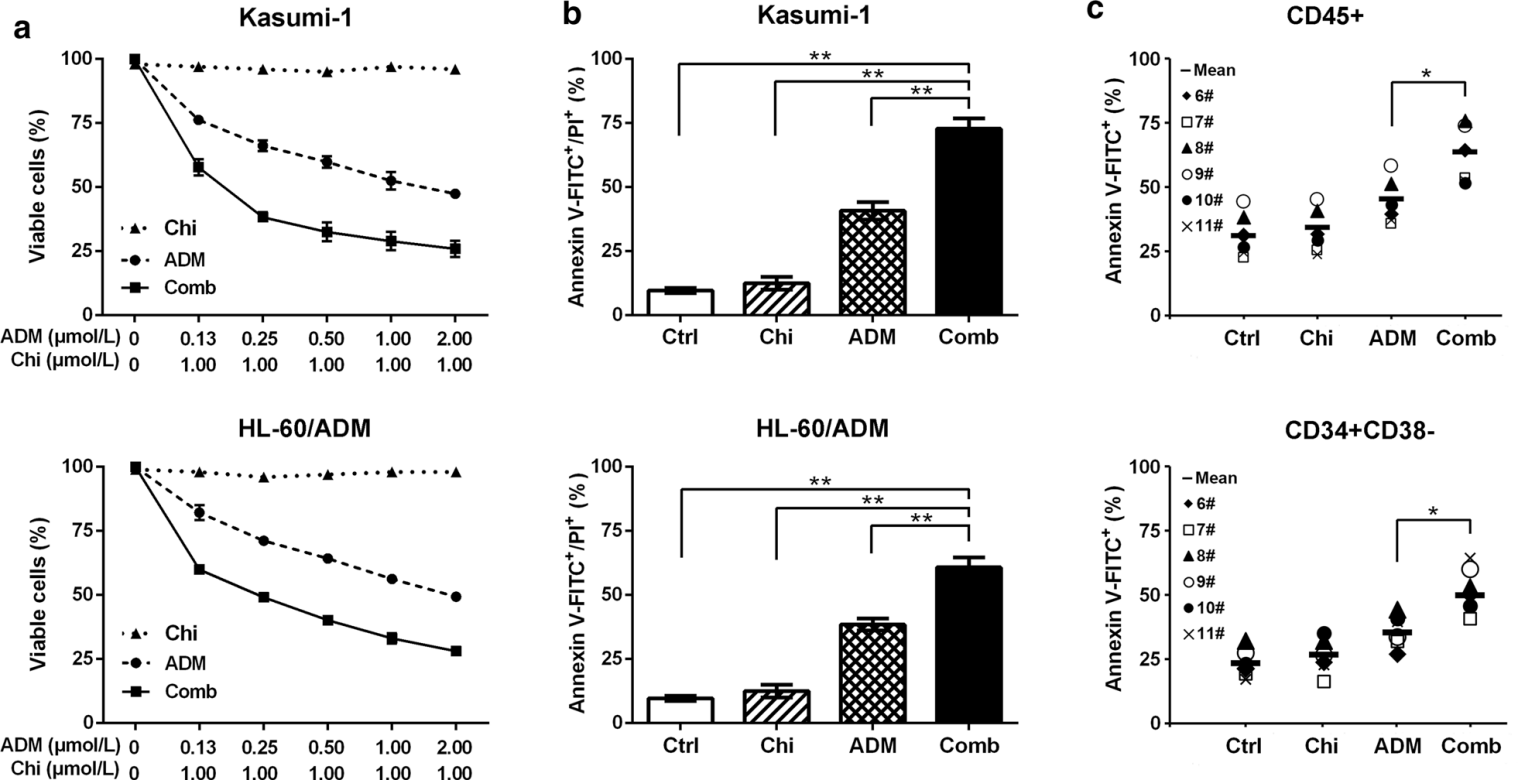
Chidamide sensitizes AML cells and stem/progenitor cells to adriamycin. **a** Kasumi-1 and HL-60/ADM cells were treated with chidamide, adriamycin or both for 24 h. Cell viability was assessed with CCK-8. **b** Kasumi-1 and HL-60/ADM cells were treated with chidamide (1.00 μmol/L), adriamycin (0.13 μmol/L) or both for 48 h. Apoptosis was determined by flow cytometry. **c** Patient samples were treated with chidamide (1.00 μmol/L), adriamycin (0.13 μmol/L) or both for 48 h. Apoptosis in CD45 + and CD34 + CD38- cells was measured by flow cytometry. ^*^P < 0.05; ^**^P < 0.01; *Ctrl* control, *Chi* chidamide, *ADM *Adriamycin, *Comb* combination

Kasumi-1 and HL-60/ADM cells were treated with chidamide, adriamycin or their combination for 24 h. 1.00 μmol/L chidamide did not inhibit proliferation, but significantly increased the growth inhibition mediated by adriamycin (Fig. 2a), the IC50 value of which was reduced from 1.39 ± 0.24 to 0.15 ± 0.05 μmol/L for Kasumi-1 cells and from 1.79 ± 0.13 to 0.25 ± 0.02 μmol/L for HL-60/ADM cells, indicating that 1.00 μmol/L chidamide increased the sensitivity of AML cells to adriamycin. We then treated Kasumi-1, HL-60/ADM cells and primary AML blasts with 1.00 μmol/L chidamide and 0.13 μmol/L adriamycin for 48 h. Chidamide (1.00 μmol/L) alone did not have cytotoxic activity, but it significantly increased adriamycin-induced apoptosis in Kasumi-1 and HL-60/ADM cells (Fig. 2b), as well as in CD45 + cells and CD34 + CD38- stem/progenitor cells from AML patients (Fig. 2c). However, chidamide (1.00 μmol/L) did not increase adriamycin-induced apoptosis in normal CD45 + or CD34 + cells from healthy donors (Additional file 2: Fig. S2).

### Inhibition of EZH2 by chidamide exerts antileukemia activity and increases adriamycin sensitivity through Smo/Gli-1 pathway

**Fig. 3:**
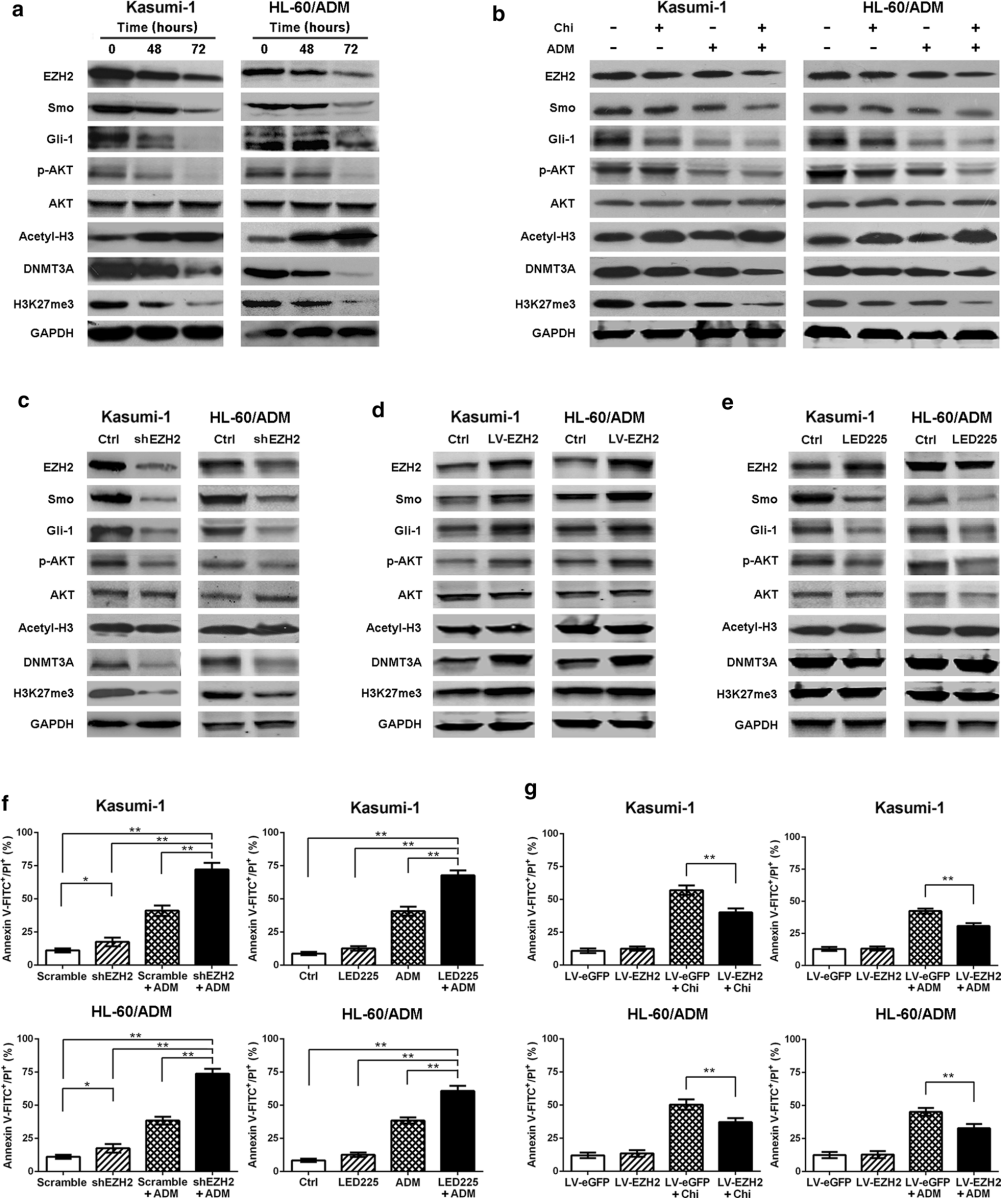
Inhibition of EZH2 by chidamide decreases the activity of Smo/Gli-1 pathway and increases sensitivity to adriamycin in AML cells. **a** Kasumi-1 and HL-60/ADM cells were treated with chidamide (10.00 μmol/L) for 48, 72 h. **b** Kasumi-1 and HL-60/ADM cells were treated with chidamide (1.00 μmol/L), adriamycin (0.13 μmol/L) or both for 48 h. **c** EZH2 was silenced with shEZH2. **d** Kasumi-1 and HL-60/ADM cells were transfected with LV-EZH2. **e** Kasumi-1 and HL-60/ADM cells were treated with LED225 (5.00 μmol/L) for 48 h. Expression of EZH2 and Smo/Gli-1 signaling and downstream targets were determined by western blotting. **f** Kasumi-1 and HL-60/ADM cells with nontarget scrambled sequence (Scramble) or shEZH2 were treated with adriamycin (0.13 μmol/L) for 48 h. Kasumi-1 and HL-60/ADM cells were treated with LED225 (5.00 μmol/L), adriamycin (0.13 μmol/L) or both for 48 h. **g** Kasumi-1 and HL-60/ADM cells with LV-eGFP or LV-EZH2 were treated with chidamide (20.00 μmol/L) or adriamycin (0.13 μmol/L) for 48 h. Apoptosis was assessed by flow cytometry. ^*^P < 0.05; ^**^P < 0.01; *Ctrl* control, *Chi* chidamide, *shEZH2* EZH2 shRNA, *LV-EZH2* EZH2-overexpressing lentivirus, *LV-eGFP* lentivirus vector carrying enhanced green fluorescent protein, *ADM* adriamycin

To understand the mechanisms of action, we treated Kasumi-1 and HL-60/ADM cells with chidamide, adriamycin or their combination and determined the protein levels after treatment for 48 or 72 h by western blotting. Chidamide (10.00 μmol/L) resulted in the accumulation of acetylated histone 3 and decreased the levels of H3K27 trimethylation (H3K27me3) and DNMT3A in Kasumi-1 and HL-60/ADM cells (Fig. 3a). We discovered that 10.00 μmol/L chidamide inhibited the expression of EZH2, the activity of Smo/Gli-1 pathway and downstream signaling target p-AKT after treatment for 48 h, and the targeted inhibition was even more effective after 72 h of treatment (Fig. 3a). This result indicated that 10.00 μmol/L chidamide inhibited the expression of EZH2 and downstream targeted trimethylation of H3K27 and DNMT3A. Interestingly, chidamide decreased the activity of Smo/Gli-1 pathway, coinciding with the potential inhibition of EZH2 expression in AML cells. Moreover, 1.00 μmol/L chidamide slightly suppressed EZH2 and p-AKT expression and Smo/Gli-1 pathway activity after treatment for 48 h, and the inhibitory effects were more obvious after combination with adriamycin in Kasumi-1 and HL-60/ADM cells (Fig. 3b). This suggests that chidamide may inhibit the Smo/Gli-1 pathway through disruption of EZH2 expression and increase the cytotoxic effect of adriamycin in AML cells.

To test this hypothesis, we silenced EZH2 with shRNA or overexpressed EZH2 in Kasumi-1 and HL-60/ADM cells. We found that genetic inhibition of EZH2 suppressed the Smo/Gli-1 pathway and downstream signaling molecule p-AKT, in addition to decreasing the levels of H3K27me3 and DNMT3A (Fig. 3c). EZH2 overexpression increased these signaling activities and the expression of downstream targets (Fig. 3d). Moreover, Smo inhibitor LED225 abrogated the activities of Smo/Gli-1 pathway, but had no effect on the levels of EZH2, H3K27me3 and DNMT3A (Fig. 3e). We also observed that shEZH2 and LED225 each increased the sensitivity to adriamycin (Fig. 3f) and that EZH2 overexpression decreased apoptosis induction by 20.00 μmol/L chidamide or 0.13 μmol/L adriamycin (Fig. 3g), although apoptosis was slightly induced after EZH2 silencing or overexpression in Kasumi-1 and HL-60/ADM cells (Fig. 3f, g). These data indicated that inhibition of EZH2 either pharmacologically by chidamide or genetically by shEZH2 decreased the activity of Smo/Gli-1 pathway and increased adriamycin sensitivity, and genetic EZH2 overexpression reduced the cytotoxicity of chidamide and restored the resistance to adriamycin in AML cells.

### Chidamide suppresses EZH2 and Smo/Gli-1 signaling and enhances the antileukemia activity of adriamycin in an AML xenograft mouse model

**Fig. 4:**
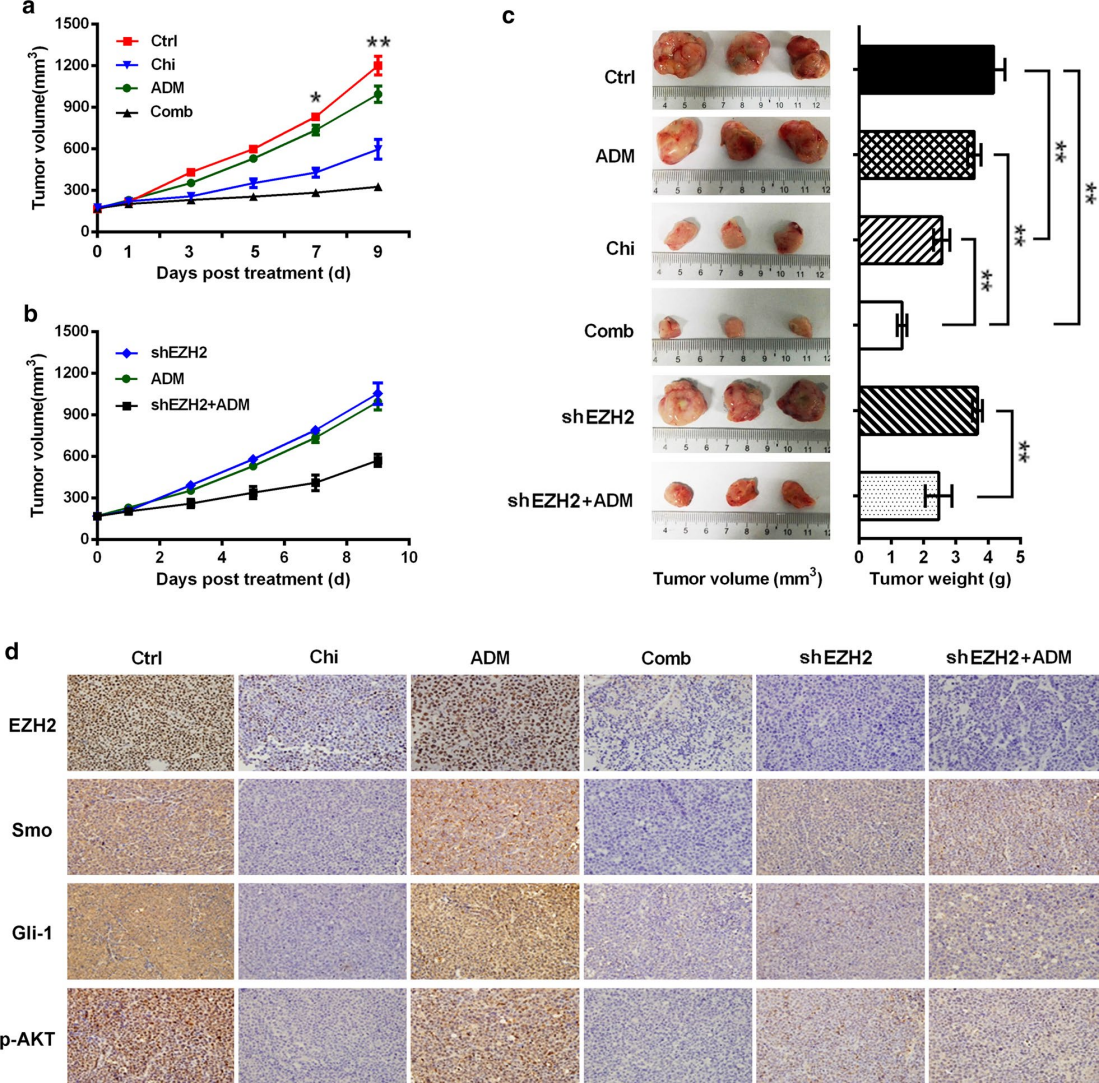
Chidamide enhances the antileukemia activity of adriamycin in leukemia-bearing mouse models. **a** Kasumi-1 tumor-bearing mice were treated with chidamide, adriamycin or both for 7 days. **b** shEZH2 Kasumi-1 tumor-bearing mice were treated with adriamycin for 7 days. **c** Tumor sizes. Tumor volume and weight were measured to assess leukemia burden in mouse models. **d** Expression of EZH2 and Smo/Gli-1 signaling were determined by histopathological and immunohistochemical examinations in leukemic tumor tissues. ^*^P < 0.05; ^**^P < 0.01; *Ctrl* control, *Chi* chidamide, *shEZH2* EZH2 shRNA, *ADM* Adriamycin, *Comb* combination

We used a leukemia-bearing mouse model to test the in vivo antileukemic and chemosensitizing activities of chidamide in AML. In our study, Kasumi-1 cells were subcutaneously implanted into BALB/c nude mice to establish an AML xenograft mouse model. Treatment with adriamycin or chidamide inhibited leukemia growth, as measured by tumor volume and weight in the mouse models, and the combination of adriamycin and chidamide was the most effective strategy in this regard (Fig. 4a and c). These results indicated that chidamide increased the antileukemia activity of adriamycin in leukemia-bearing mice. To further demonstrate that the effect of chidamide on chemosensitivity in vivo was mediated in part through EZH2 inhibition, Kasumi-1 cells transfected with shEZH2 were subcutaneously implanted to establish a leukemia-bearing mouse model. We observed that depletion of EZH2 increased the antileukemia activity of adriamycin in the mouse models (Fig. 4b, c). Histopathological and immunohistochemical examinations showed that chidamide decreased the expression of EZH2 and inhibited the Smo/Gli-1 pathway and downstream signaling molecule p-AKT (Fig. 4d). Genetic inhibition of EZH2 also suppressed the Smo/Gli-1 signaling pathway in leukemic tumor tissues (Fig. 4d). These in vivo data suggested that EZH2 inhibition by chidamide or shEZH2 decreased leukemia growth and increased the antileukemia effect of adriamycin through suppression of Smo/Gli-1 pathway in the leukemia-bearing mouse models.

## Discussion

**Fig. 5:**
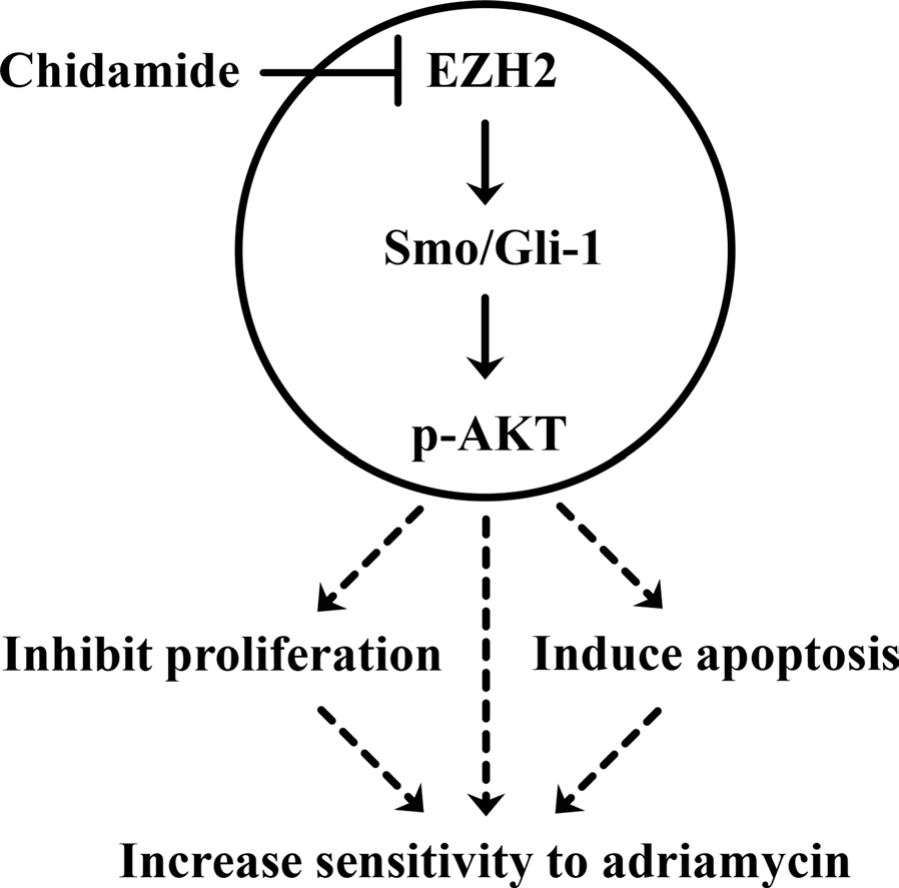
Model for the possible mechanism of chidamide-mediated chemosensitization in AML cells. Disruption of EZH2 by chidamide inhibited proliferation, induced apoptosis and increased the sensitivity to adriamycin through Smo/Gli-1 pathway in AML cells

In this study, we investigated the antileukemia activity of HDAC inhibitor chidamide alone and in combination with adriamycin in AML cells in vitro and in vivo. We discovered that chidamide suppressed growth, induced apoptosis and increased sensitivity to adriamycin in AML cells and stem/progenitor cells by inhibiting EZH2. We further demonstrated that in addition to decreasing the levels of H3K27me3 and DNMT3A, pharmacological or genetic inhibition of EZH2 decreased the activity of Smo/Gli-1 pathway and increased adriamycin sensitivity in AML cells (Fig. 5).

HDACs play an essential role in the development of leukemia, and combination of HDAC inhibitor with chemotherapeutic drug is a potentially effective approach against AML [45–48]. Our studies demonstrated that chidamide had antileukemia activity and sensitized AML cells to adriamycin. In an effort to understand the mechanism of action, we found that inhibition of EZH2 by chidamide or shEZH2 decreased the levels of H3K27me3 and DNMT3A and increased the cytotoxic activity of adriamycin, and EZH2 overexpression had the opposite effects on AML cells. EZH2 is a histone methyltransferase associated with transcriptional repression through H3K27me2/3, and EZH2-mediated H3K27 methylation is closely related to pathological processes and poor prognosis in hematological malignancies [49, 50]. Inhibition of EZH2 suppresses the level of H3K27me3, induces leukemia cell apoptosis and depletes LSCs [41, 51]. EZH2 controls CpG methylation by directly contacting with DNMTs in PRC2/3 and is related to the activities of DNMT1, DNMT3A, and DNMT3B [49, 52]. DNMT1- and EZH2-mediated methylation contributes to the progression of gastric cancer and glioblastoma [53]. Combined inhibition of EZH2 and DNA methylation produces a synergistic antileukemia effects through epigenetic regulation of multiple genes expression [54]. HDAC inhibitors decrease the expression of EZH2 and DNMT1 and increase hypomethylating agent-mediated apoptosis in human leukemia cells [55–57]. Knockdown of EZH2 expression inhibits histone methyltransferase activity, reduces H3K27me2/3 levels and increases the inhibition of clonogenic survival mediated by HDAC inhibitor in AML cells [58]. Combined inhibition of EZH2 and HDAC increases the depletion of PRC2 complex proteins and synergistically induces apoptosis in cultured and primary AML cells [59]. A first-in-class dual EZH2/HDAC inhibitor has been biologically investigated in AML cells, and its antileukemia activity is associated with proliferation inhibition, apoptosis induction and cell cycle arrest [60]. Therefore, targeted inhibition of EZH2 and HDACs has been developed as an epigenetic therapeutic strategy against AML.

In this study, we demonstrated that EZH2 inhibition by chidamide disrupted the activity of Smo/Gli-1 pathway and downstream signaling molecule p-AKT and increased the adriamycin sensitivity of AML cells and stem/progenitor cells. Mechanistic studies showed that depletion of EZH2 by shRNA decreased the levels of H3K27me3 and DNMT3A and inhibited the activity of Smo/Gli-1 pathway, while disruption of Smo/Gli-1 pathway did not affect EZH2 expression. This result indicated that inhibition of EZH2 or Smo decreased the activity of Smo/Gli-1 pathway and contributed to the increasing sensitivity to adriamycin in AML cells. The Smo/Gli-1 pathway plays critical roles in embryogenesis and developmental processes, and its activity is essential for chemoresistance and maintenance of LSCs in myeloid leukemia [61–63]. Studies have shown that disruption of Smo/Gli-1 signaling increases chemosensitivity through downregulation of IGF-1R/Akt/MRP1 pathway and transcriptional control of twist1 and snail in AML cells [42, 64]. Smo inhibitors improve therapeutic efficacy by sensitizing dormant LSCs to chemotherapy and overcoming microenvironment-induced chemoresistance, and targeting Gli-1 also suppresses proliferation and enhances chemosensitivity in AML cells and progenitor cells [65–67]. Clinical studies have demonstrated that in combination chemotherapy, Smo inhibitor glasdegib benefits AML patients by increasing overall survival in the absence of complete remission (CR), suggesting that the antileukemia activity of glasdegib may be mediated through elimination of LSCs [68–70]. A recent study shows that the activity of Hedgehog pathway is largely independent of Smo and therefore inherently resistant to Smo inhibitors in AML, and hypomethylating agent improves glasdegib sensitivity by increasing the level of Gli-3 repressor and modulation of Gli-1 [71]. HDAC inhibitors abrogate Smo-dependent and Smo-independent GLI activation and Hedgehog-targeted gene expression and overcome drug resistance to Smo inhibitors in cancer cells [72, 73]. Combinatorial inhibition of HDACs and Hedgehog pathway synergistically suppresses tumor growth and homologous recombination in aerodigestive cancers, and a dual-targeted inhibitor is capable of effectively overcoming the Smo inhibitor resistance conferred by Smo mutations [74, 75]. Moreover, EZH2 inhibition results in multitarget-mediated suppression of Hedgehog pathway, increases chemosensitivity and decreases self-renewal in colorectal cancer-initiating cells [33, 40]. This finding supports the rational combination of HDAC inhibitor and chemotherapy for the treatment of AML.

## Conclusions

In our study, disruption of EZH2 by chidamide was demonstrated to inhibit proliferation, induce apoptosis and improve sensitivity to adriamycin through disruption of Smo/Gli-1 pathway in AML cells. This study provides a promising strategy for the combination of HDAC inhibitors and cytotoxic drugs to improve chemotherapeutic effects in AML.

## Supplementary Information

**Additional file 1**:** Fig. S1**. Chidamide has limited cytotoxicity in normal CD45+ and CD34+ cells. Bone marrow samples from healthy donors were treated with chidamide for 48 hours. Apoptosis in CD45+ and CD34+ cells was determined by flow cytometry.

**Additional file 2**:** Fig. S2**. Chidamide doesn’t sensitize normal CD45+ and CD34+cells to adriamycin. Bone marrow samples from healthy donors were treated with chidamide (1.00 μmol/L), adriamycin (0.13 μmol/L) or both for 48 hours. Apoptosis in CD45+ and CD34+ cells was determined by flow cytometry. Ctrl, control; Chi, chidamide; ADM, adriamycin; Comb, combination.

## Data Availability

The dataset supporting the conclusions of this article is included within the article.

## Abbreviations

AML: Acute myeloid leukemia
LSC: Leukemia stem cell
HAT: Histone acetyltransferase
HDAC: Histone deacetylase
EZH2: Enhancer of zeste homolog 2
FDA: Food and Drug Administration
RT-PCR: Real-time polymerase chain reaction
