# Supplementary figures and images for: Inhibition of EZH2 by chidamide exerts antileukemia activity and increases chemosensitivity through Smo/Gli-1 pathway in acute myeloid leukemia
Source: J Transl Med. 2021 Mar 21;19:117. doi: 10.1186/s12967-021-02789-3 (PMC7981995; doi:10.1186/s12967-021-02789-3)

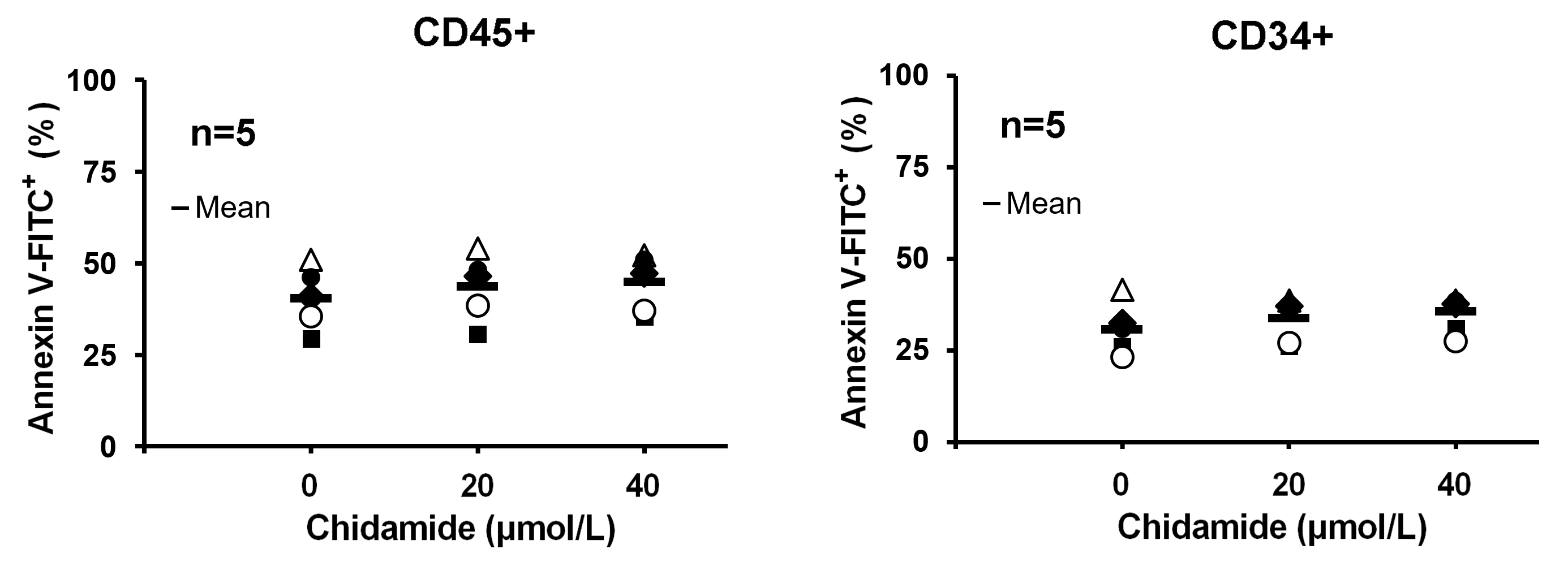

Supplement: Supplementary file 1 — Additional file 1: Fig. S1. Chidamide has limited cytotoxicity in normal CD45+ and CD34+ cells. Bone marrow samples from healthy donors were treated with chidamide for 48 hours. Apoptosis in CD45+ and CD34+ cells was determined by flow cytometry. [file 12967_2021_2789_MOESM1_ESM.tif]

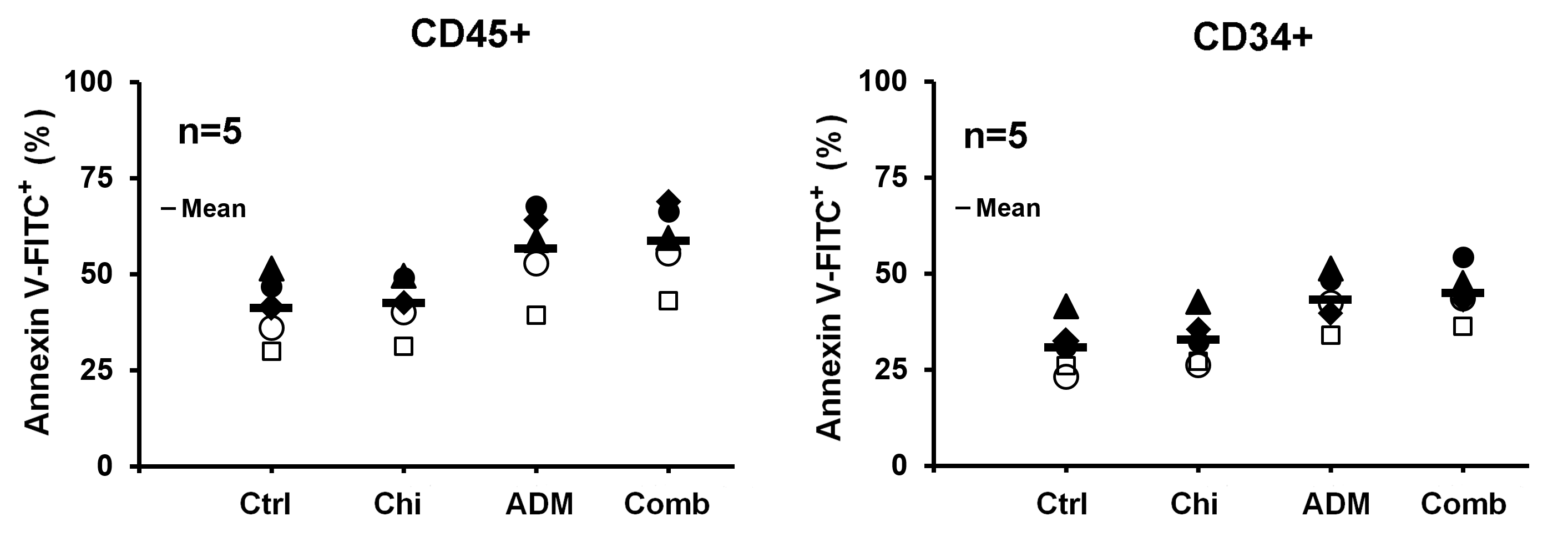

Supplement: Supplementary file 2 — Additional file 2: Fig. S2. Chidamide doesn’t sensitize normal CD45+ and CD34+cells to adriamycin. Bone marrow samples from healthy donors were treated with chidamide (1.00 μmol/L), adriamycin (0.13 μmol/L) or both for 48 hours. Apoptosis in CD45+ and CD34+ cells was determined by flow cytometry. Ctrl, control; Chi, chidamide; ADM, adriamycin; Comb, combination. [file 12967_2021_2789_MOESM2_ESM.tif]
